# The prevalence and correlates of sitting in European adults - a comparison of 32 Eurobarometer-participating countries

**DOI:** 10.1186/1479-5868-10-107

**Published:** 2013-09-11

**Authors:** Jason A Bennie, Josephine Y Chau, Hidde P van der Ploeg, Emmanuel Stamatakis, Anna Do, Adrian Bauman

**Affiliations:** 1 Prevention Research Collaboration, Sydney School of Public Health, The University of Sydney, 92-94 Parramatta Road, Camperdown, NSW 2050, Australia; 2 Department of Public and Occupational Health, EMGO Institute for Health and Care Research, VU University Medical Centre, Van der Boechorststraat 7, Amsterdam, Netherlands; 3 Physical Activity Research Group (UCL-PARG), Division of Population Health, Department of Epidemiology and Public Health, University College London, 4th Floor, Holborn Union Building Archway (Whittington) Campus, Highgate Hill, London N19 5LW, UK; 4 Centre for Epidemiology and Evidence, New South Wales Ministry of Health, Locked Mail Bag 961, North Sydney, NSW 2059, Australia

**Keywords:** Sitting, Adults, Europe, Prevalence, Correlates

## Abstract

**Background:**

Prolonged sitting is an emerging health risk. However, multi-country comparative sitting data are sparse. This paper reports the prevalence and correlates of sitting time in 32 European countries.

**Methods:**

Data from the Eurobarometer 64.3 study were used, which included nationally representative samples (n = 304-1,102) from 32 European countries. Face-to-face interviews were conducted during November and December 2005. Usual weekday sitting time was assessed using the International Physical Activity Questionnaire (short-version). Sitting time was compared by country, age, gender, years of education, general health status, usual activity and physical activity. Multivariable-adjusted analyses assessed the odds of belonging to the highest sitting quartile.

**Results:**

Data were available for 27,637 adults aged 15–98 years. Overall, mean reported weekday sitting time was 309 min/day (SD 184 min/day). There was a broad geographical pattern and some of the lowest amounts of daily sitting were reported in southern (Malta and Portugal means 194–236 min/day) and eastern (Romania and Hungary means 191–276 min/day) European countries; and some of the highest amounts of daily sitting were reported in northern European countries (Germany, Benelux and Scandinavian countries; means 407–335 min/day). Multivariable-adjusted analyses showed adults with low physical activity levels (OR = 5.10, CI_95_ = 4.60-5.66), those with high sitting in their main daily activity (OR = 2.99, CI_95_ = 2.74-3.25), those with a bad/very bad general health state (OR = 1.87, CI_95_ = 1.63-2.15) and higher education levels (OR = 1.48, CI_95_ = 1.38-1.59) were more likely to be in the highest quartile of daily sitting time. Adults within Greece (OR = 2.91, CI_95_ = 2.51-3.36) and Netherlands (OR = 2.56, CI_95_ = 2.22-2.94) were most likely to be in the highest quartile. High-sit/low-active participants comprised 10.1% of the sample. Adults self-reporting bad/very bad general health state (OR = 4.74, CI_95_ = 3.97-5.65), those within high sitting in their main daily activities (OR = 2.87, CI_95_ = 2.52-3.26) and adults aged ≥65 years (OR = 1.53, CI_95_ = 1.19-1.96) and were more likely to be in the high-sit/low-active group.

**Conclusions:**

Weekday sitting time and its demographic correlates varied considerably across European countries, with adults in north-western European countries sitting the most. Sitting is prevalent across Europe and merits attention by preventive interventions.

## Background

Sedentary behaviour (too much sitting as opposed to too little exercise) has recently emerged as a candidate independent risk factor associated with several detrimental health outcomes [1,2]. Sedentary behaviours are defined as any waking behaviour characterized by an energy expenditure ≤1.5 METs while in a sitting or reclining posture [3,4]. High volumes of time spent sitting or engaged in sedentary behaviour have been associated with an increased risk of all-cause mortality [5-7] and risk of developing type 2 diabetes [8], obesity [9-11] and cardiovascular disease [12-15]. Importantly, in some studies these associations with mortality and health have occurred irrespective of whether an individual meets the core adult physical activity recommendation of 150 minutes of at least moderate-intensity physical activity per week [5,7,16-18]. Several international health authorities have recently provided formal recommendations citing the importance of reducing sitting time [19-21].

Until recently, public health surveillance has primarily focused on understanding the distribution of population leisure-time physical activity (e.g. structured moderate-to-vigorous-intensity exercise) [22,23] and active travel (e.g. walking and cycling for transportation) [24,25]. The lack of population studies assessing sitting time was recently highlighted by the recent Lancet Series on physical activity [26]. One of the potential reasons for the insufficient population data on sitting time may be that it has been considered as a chronic disease risk factor for less than a decade [27]. Moreover, until recently, there has been a lack of consistency in measurement instruments used to assess sitting time and hence it is difficult to compare sitting time across countries [28]. The International Physical Activity Questionnaire (IPAQ) assesses self-reported sitting time. It has been shown to have acceptable reliability and validity [29,30] and is therefore applicable for use in population studies. The IPAQ has two versions, a long and short version, and each have recently been used in several large-scale multi-country physical activity surveillance studies [28,31,32].

The International Prevalence Study (IPS) of physical activity is one of the few population studies that examined the prevalence and correlates of sitting time concurrently across several countries [28]. In this 20 country comparison, the mean daily weekday sitting time, assessed using the IPAQ short version, for the overall sample (N = 49,493; age range 18–65 years) was 346 minutes/day [28]. These sitting times differed from smaller scale single-country studies that have used the IPAQ sitting time question. For example, in a study of 1,200 Belgium adults (aged 20–65 years), mean sitting time was 421 minutes/day [33]. Additionally, a study of 2,000 German adults (aged 49.3 ± 17.3 years) using the Global Physical Activity Questionnaire identified that median daily sitting time was 300 minutes/day [34]. Even though the IPS used a harmonized instrument, there were considerable between-country variations in sitting time [28]. For example, adults within Japan and Saudi Arabia reported approximately double the median daily sitting (~360 minutes per/day) than those from Portugal and Brazil (~180 minutes/day). Furthermore, the IPS study identified that high volumes of sitting were more prevalent among younger adults (18–39 years) and those with greater than 13 years of education [28].

Examining how sitting time differs across levels of physical activity may also warrant attention. For example, within populations there may be sub-groups who engage in disparate patterns of sitting and physical activity. Of particular interest for public health surveillance may be adults classified as high-sitting/low-active, which reflects the combined risks of sitting and inactivity. Therefore, as an alternative to examining sitting and physical activity in isolation, it is of interest to examine how sitting time differs according to physical activity levels. However, at present few studies have examined how sitting and physical activity levels differ within populations and within different sociodemographic groups and across countries.

There is a need to report cross-country population sitting data [26]. Such research is important because this will provide international and national public health policy and health services with current sitting data for tracking trends and geographical patterns of sitting time, identifying ‘at risk’ populations, and informing evidence-based approaches to reducing sitting.

The primary aim of this study is to describe the prevalence and correlates of daily sitting time among a large representative sample of adults from 32 European countries, and to assess between and within country variations. A secondary aim is to examine the prevalence and correlates of high-sit/low-active populations within the sample.

## Methods

### Recruitment and participants

Data were drawn from the Eurobarometer 64.3 (EB 64.3). The Eurobarometer survey series, running since 1970, is a cross-national longitudinal study designed to compare and gauge trends within Europe. The Eurobarometer survey is typically carried out each autumn and spring [35]. The Eurobarometer 64.3 was conducted between November-December 2005 and assessed a number of factors, including health questions. Field work for the study was carried out by a consortium of market and public opinion research agencies, requested by the European Commission, Directorate-General Press and Communication, Opinion Polls (see http://www.icpsr.umich.edu/icpsrweb/ICPSR/studies/4590 for further information on EB 64.3). Data from the Eurobarometer survey series are publicly accessible. With the exception of using the data for commercial purposes, the reproduction of Eurobarometer data is authorized [35]. The applicable EB 64.3 survey methods are described in at http://ec.europa.eu/public_opinion/index_en.htm. The European Commission approved the protocols and written informed consent from all participants was obtained [35]. Eurobarometer 64.3 utilized a multistage random sampling design in all countries, and all interviews were conducted face-to-face in people’s homes, in the national language. Computer-assisted personal interview (CAPI) was used in countries where that technique was available and, where unavailable, paper-based surveys were conducted [35]. Sample sizes within countries ranged from 304 (Northern Ireland) to 1,102 (France), with a total of 29,131 participants. The average response rate across countries for the face-to-face interviews was 54.6% [35].

### Measures and data management

The IPAQ short version was used to assess usual weekday sitting and physical activity [29,30]. For sitting time, a question was asked as follows: *During the last seven days, how much time did you usually spend sitting on a weekday?* The weekday was chosen to reflect habitual behaviour for the short IPAQ instrument (http://www.ipaq.ki.se), which has been identified to have acceptable reliability and validity for assessing usual sitting time [30]. The sitting data were truncated at 960 minutes/day (16 hours), under the assumption that an otherwise healthy ambulatory adult would be mobile for at least 8 hrs each day (e.g. light-intensity walking from place to place, around the house, at work etc.). A total of 68 out of 27,637 cases (0.2%) were truncated for reporting sitting >960 minutes/day. As shown in Table 1, the mean, median and interquartile range of daily sitting times were generally concordant across categories. Therefore, to aid interpretation of results, we report these sitting data as means of usual weekday sitting time. The sitting data were also presented in quartiles: 1) 0–179; 2) 180–299; 3) 300–419; and 4) 420–960 mins/day.

**Table 1:** Mean, median and interquartile range for Eurobarometer 64.3 IPAQ sitting time in minutes by selected sociodemographic characteristics and country

	**n**	**Mean (SD)**	***p-value***	**Median (Interquartile range)**
**All**	*27 637*	309 (±185)	**–**	300 (180–420)
**Gender**		**Mean (95% CI)**		
Male	*12 234*	320 (316–323)	<0.001*	300 (180–480)
Female	*15 403*	301 (298–304)		270 (180–420)
**Age**		**Mean (95% CI)**		
15 – 24 years	*3 764*	363 (357–369)	<0.001**	360 (240–480)
25 – 34 years	*4 496*	306 (301–312)		270 (180–420)
35 – 44 years	*4 931*	293 (289–298)		240 180–420)
45 – 54 years	*4 506*	301(296–307)		240 180–420)
55 – 64 years	*4 409*	289 (284–294)		240 180–420)
65 years and older	*5 531*	313 (309–318)		300 (180–360)
**Education**		**Mean (95% CI)**		
18 yrs and Less	*15 100*	279 (276–282)	<0.001**	240 (180–360)
19 years and over	*9 277*	334 (331–338)		300 (180–480)
**Physical activity level**		**Mean (95% CI)**		
Low-active	*7 590*	340 (335–345)	<0.001**	300 (180–420)
Moderate	*13 648*	322 (319–325)		300 (180–427)
High-active	*6 399*	247 (244–251)		240 (120–320)
**General state of health**		**Mean (95% CI)**		
Very good	*5 750*	318 (313–322)	<0.001**	300 (180–465)
Good	*13 826*	307 (304–310)		300 (180–420)
Neither good nor bad	*5 878*	298 (293–303)		270 (180–420)
Bad/very bad	*2 108*	336 (327–345)		300 (180–480)
**Usual activity**^**a**^		**Mean (95% CI)**		
Low sitting	*8 104*	245 (241–248)	<0.001**	240 (120–300)
Mixed sitting	*10 103*	331 (328–335)		300 (180–480)
High sitting	*9 430*	342 (338–336)		300 (180–480)
**Country**		**Mean (95% CI)**		
The Netherlands	*1 011*	407 (395–420)	<0.001**	360 (240–540)
Denmark	*977*	383 (371–395)		360 (240–480)
Czech Republic	*943*	375 (363–388)		345 (240–510)
Greece	*996*	374 (364–384)		360 (240–480)
Cyprus (Republic)	*467*	359 (342–377)		360 (180–480)
Belgium	*992*	342 (330–353)		300 (180–480)
Sweden	*1 030*	340 (330–351)		300 (180–480)
Cyprus (TCC)	*415*	338 (321–353)		300 (240–420)
Germany West	*894*	335 (324–347)		300 (180–450)
Finland	*979*	335 (332–347)		300 (180–480)
Estonia	*950*	334 (323–345)		300 (180–480)
Poland	*964*	333 (321–345)		300 (180–480)
Great Britain	*900*	326 (314–339)		300 (180–420)
Luxembourg	*492*	316 (300–333)		300 (180–420)
Slovakia	*982*	314 (304–325)		300 (180–420)
Germany East	*530*	314 (300–327)		300 (180–420)
Slovenia	*1 005*	312 (301–323)		270 (180–450)
Austria	*1 002*	309 (299–319)		300 (180–420)
Croatia	*978*	308 (297–320)		270 (180–450)
Turkey	*875*	305 (294–316)		270 (180–360)
Northern Ireland	*286*	303 (283–322)		240 (180–360)
Bulgaria	*925*	298 (290–308)		240 (180–360)
Ireland	*894*	284 (274–295)		240 (180–360)
Spain	*949*	284 (274–294)		244 (179–366)
France	*976*	282 (271–293)		240 (180–360)
Latvia	*1 000*	272 (260–283)		240 (120–420)
Hungary	*969*	267 (256–277)		240 (150–360)
Italy	*960*	266 (256–276)		240 (120–360)
Lithuania	*872*	263 (251–275)		240 (120–360)
Malta	*444*	236 (222–251)		210 (120–360)
Portugal	*1 000*	194 (184–204)		180 (60–300)
Romania	*980*	191 (179–203)		135 (0–300)

*T-test of significance between male and female.

**ANOVA with Scheffěs post hoc tests for significance between age, occupation, physical activity level, general state of health & country.

^a^Low Sit usual activities = responsible for ordinary shopping, unemployed, temporarily not working, unskilled manual worker, fisherman, skilled manual worker and farmer, Mixture = student, retired, unable to work, supervisor, High sit = employed position, at desk, general management, employed professional middle management, professional employed travelling position, owner of a shop, craftsmen, service job.

Physical activity was assessed using six items in the IPAQ short version, which asked about frequency and duration of vigorous intensity, moderate intensity and walking physical activity. The questionnaire was scored using established methods (http://www.ipaq.ki.se). We classified participants into three levels of physical activity: high-, moderate-, and low-active groups. The activity category ‘high’ given to participants who met either of the following two criteria: a) vigorous-intensity activity on at least 3 days achieving a minimum total physical activity of at least 1500 MET-minutes/week; or b) 7 or more days of any combination of walking, moderate-intensity or vigorous-intensity activities achieving a minimum total physical activity of at least 3000 MET-minutes/week. The pattern of activity to be classified as ‘moderate’ was meeting either of the following three criteria: a) 3 or more days of vigorous-intensity activity of at least 20 minutes per day; b) 5 or more days of moderate-intensity activity and/or walking of at least 30 minutes per day; or c) 5 or more days of any combination of walking, moderate-intensity or vigorous intensity activities achieving a minimum total physical activity of at least 600 MET-minutes/week. Participants who did not meet criteria for categories ‘moderate’ or ‘high’ were considered to have a ‘low’ physical activity level (http://www.ipaq.ki.se). These physical activity and sitting time assessments have been shown to have acceptable reliability and validity [29,30].

Usual daily sitting time was compared across the 32 participating European Union countries. To examine whether sitting time varied across sociodemographic factors, sitting data were compared across the following explanatory variables: gender; age in six categories (15–24, 25–34, 35–44, 45–54, 55–64, and 65 yrs and older); years of education in two categories (18 yrs and less, and 19 yrs and over); physical activity level in three categories, (low-, moderate-, and high-active); self-reported general state of health in four categories (very good, good, neither good nor bad and bad/very bad); and main activity in three categories:1) low sitting occupations (responsible for ordinary shopping, unemployed, unskilled manual worker, fisherman, skilled manual worker and farmer); 2) mixed sitting occupations (student, retired, unable to work, supervisor); and 3) high sitting occupations (employed position at desk, general management, employed professional middle management, professional employed travelling position, owner of a shop, craftsmen and service job).

We examined the distribution of sitting time across sociodemographic factors and countries among specific sub-groups at the highest/lowest risk based on physical activity and sitting time. For these analyses, we used the three physical activity categories described above: low-, moderate-, and high-active. In parallel, the sitting data was divided into three groups based on quartiles of sitting time: low-sit (lowest quartile: 0–211 minutes/day); medium-sit (quartiles two and three combined: 211–419 minutes/day); and high-sit (highest quartile: 420–960 minutes/day). The information from physical activity level and sitting time was used to categorise the sample as: 1) high-sit/low-active; 2) low-sit/high-active; and 3) neither high-sit/low-active nor low-sit/high-active. A total of 2,779 out of 27,637 (10.1% of the total sample) were classified as high-sit/low-active, and 3,134 (11.3%) were categorised as low-sit/high-active, leaving a total of 21,742 (78.6%) participants classified as neither high-sit/low-active nor low-sit/high-active.

### Statistical analysis

Data analyses were conducted using SAS software, Version 9.3 of the SAS System for Windows. Data on sitting were analysed using parametric analyses for normally distributed data. Sitting data were presented as means and medians for usual daily sitting time with 95% confidence intervals (CI). Also, the interquartile range and quartiles of sitting time were reported to reflect increasing categories of sitting time. Independent t-tests were used to assess whether sitting time differed by gender and by education level. A one-way ANOVA was performed with Scheffěs post-hoc test to examine whether sitting time differed between levels of age, occupational category, physical activity level, general health and country. A Pearson’s chi-squared test examined whether there were differences by country and selected sociodemographic factors among participants classified within the high-sit/low-active or low-sit/high-active sub-groups. A significance level (alpha) for the one-way ANOVA, independent t-tests and Pearson’s chi-squared test was 0.05.

A logistic regression analysis was used to assess the odds of belonging to the highest quartile of sitting time. Adjusted odds ratios, with 95% confidence intervals, were reported for these analyses. The response variable was dichotomous and indicated if a participant belonged to the highest quartile of sitting time (420–960 minutes/day) or quartiles one to three of sitting time (0–419 (minutes/day). The model included the explanatory variables: gender (reference = “Male”); education level (reference = “18 years and less”); age (reference = “15-24 years”); occupation category (reference = “low-sitting”); physical activity level (reference = “high-active”); general state of health (reference = “very good”) and country (relative to the mean odds for all countries analysed). In separate logistic regression models, we assessed the odds of belonging to the “high-sit/low-active” group and the odds of belonging to the “low-sit/high-active” group. Except for physical activity level, the model included the same explanatory variables used in the previous logistic regression analyses.

## Results

Information on the Eurobarometer 64.3, sampling and response rates have been described [35]. In brief, data were available for 29,193 adults aged 15–98 years from 32 European countries. For the sitting time question, data were missing for 1,556 participants (5.3% of total sample) and, therefore, the analyses included 27,637 participants.

Descriptive data on usual weekday sitting time are presented for the total sample, by country and selected sociodemographic factors (Table 1). Significant differences were observed within each sub-group across all sociodemographic factors and by country. Mean weekday sitting time for the total sample was 309 minutes/day (SD = 185 minutes/day), equating to 5–6 hours of sitting per day. Males reported a higher mean weekday sitting time than females, and younger people (15–24 years) had higher sitting times when compared to older people (≥25 years). Participants with ≤18 years of education reported lower weekday sitting times than adults with ≥19 years of education. There was an inverse relationship between physical activity level and usual weekday sitting time. Adults in the high-active category reported lower weekday sitting times than those in the moderate group, who reported less than those within the low-active category. Adults who reported their general health to be neither good nor bad had the lowest sitting times, whereas adults who reported having bad/very bad general health reported the highset mean sitting times. There were differences in sitting time by usual activity with those within high sitting usual activates reporting the highest sitting, followed by mixed sitting activities. There was a large variation in mean sitting times between countries. Adults from the Netherlands, Denmark, Czech Republic and Greece reported the highest mean sitting times (376–407 minutes/day), while those from Romania, Portugal, Malta and Lithuania reported the lowest mean sitting times (191–236 minutes/day) (Table 1).

**Figure 1:**
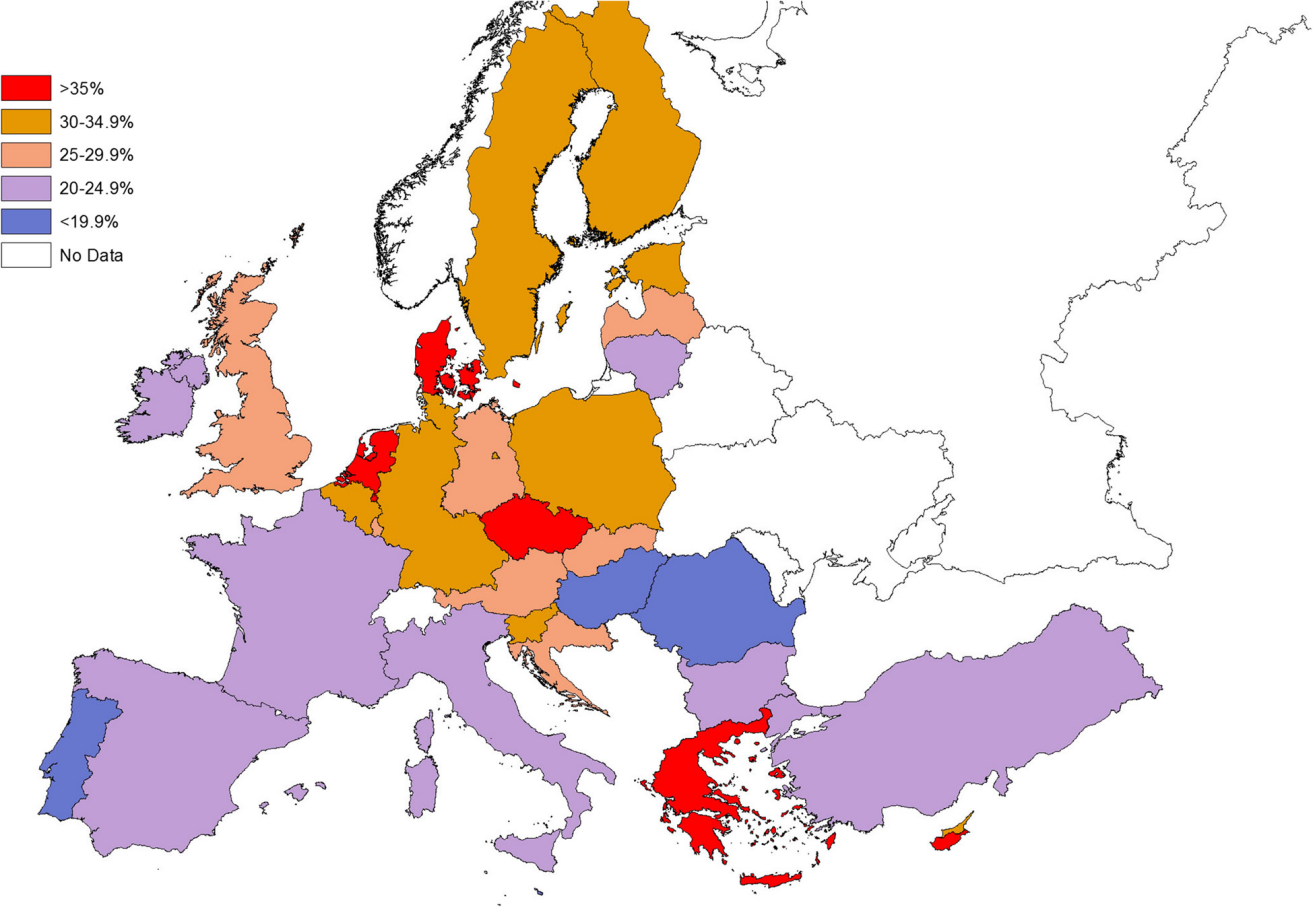
Proportion of people 15 years and older who sit for 7 or more hours per day by country.

The cross-country distributions of proportions of adults within the highest quartile of sitting time (420–960 minutes/day) are shown in Figure 1. A geographical pattern was observed with greater proportions of north-western European countries having ≥30% of the sample in the highest quartile. In contrast, countries within south-eastern Europe generally typically had the lowest proportions within the highest quartile (Figure 1).

Multivariable-adjusted analyses for the odds of being in the highest sitting quartile are shown in Table 2. Females (OR = 0.75, CI_95_ = 0.71-0.80) were less likely to be in the highest sitting quartile. Those in the low active (OR = 5.10, CI_95_ = 4.60-5.66) and moderate activity group (OR = 2.78, CI_95_ = 2.53-3.06) were more likely to be in the highest sitting quartile, compared with the high active group. Compared to adults reporting very good health, those who reported their general health as neither good nor bad (OR = 1.28, CI_95_ = 1.15-1.42) and bad/very bad (OR = 1.87, CI_95_ = 1.63-2.15) were more likely to be in the highest sitting quartile. Compared to low sit usual activities, those in high sit (OR = 2.99, CI_95_ = 2.74-3.25) and mixed sitting activities (OR = 1.36, CI_95_ = 1.21-1.52) were more likely to report high levels of sitting. Adults within Greece (OR = 2.91, CI 2.51-3.36) and the Netherlands (OR = 2.56, CI_95_ = 2.22-2.94) had the highest odds for reported high levels of sitting compared with the average odds for all countries. In contrast, those within Portugal (OR = 0.41, CI_95_ = 0.33-0.51) and Malta (OR = 0.46, CI_95_ = 0.35-0.61) were less likely to be in the highest quartile (Table 2).

**Table 2:** **Multivariable-adjusted odds ratios**^**a **^**(OR) and 95% confidence intervals (95% CI) for being in the highest sitting quartile (420–960 min/day) by sociodemographic factors and by country**

**Independent variables**	**OR (95% CI)**
**Gender (ref: male)**	
Female	0.75 (0.71–0.80)
**Age (ref: 15–24 years)**	
25–34 years	0.91 (0.78–1.06)
35–44 years	0.82 (0.71–0.96)
45–54 years	0.86 (0.74–1.00)
55–64 years	0.73 (0.62–0.86)
65 years and older	1.06 (0.89–1.26)
**Education (ref: 18 yrs and less of education)**	
19 years and over of education	1.52 (1.42–1.63)
**Physical activity Level (ref: high-active)**	
Low-active	5.10 (4.60–5.66)
Moderate	2.78 (2.53–3.06)
**General state of health (ref: very good)**	
Good	1.08 (0.99–1.18)
Neither good nor bad	1.28 (1.15–1.42)
Bad/very bad	1.87 (1.63–2.15)
**Usual activity (ref: low sitting)**	
Mixed sitting	1.36 (1.21–1.52)
High sitting	2.99 (2.74–3.25)
**Country (ref: mean odds of all countries)**	
Greece	2.91 (2.51–3.36)
The Netherlands	2.56 (2.22–2.94)
Czech Republic	1.76 (1.52–2.04)
Cyprus (Republic)	1.70 (1.37–2.11)
Denmark	1.57 (1.34–1.84)
Germany West	1.36 (1.16–1.59)
Poland	1.35 (1.15–1.58)
Finland	1.25 (1.07–1.46)
Belgium	1.22 (1.05–1.42)
Germany East	1.20 (0.96–1.49)
Estonia	1.19 (1.02–1.40)
Croatia	1.17 (0.99–1.38)
Great Britain	1.11 (0.94–1.30)
Turkey	1.09 (0.90–1.32)
Slovakia	1.05 (0.90–1.23)
Slovenia	1.04 (0.88–1.23)
Austria	1.02 (0.87–1.20)
Cyprus (TCC)	1.02 (0.81–1.29)
Bulgaria	1.00 (0.85–1.19)
Sweden	0.94 (0.81–1.09)
Luxembourg	0.90 (0.71–1.13)
Northern Ireland	0.84 (0.63–1.12)
Latvia	0.82 (0.69–0.97)
Hungary	0.73 (0.61–0.87)
Lithuania	0.67 (0.56–0.81)
Ireland	0.64 (0.53–0.78)
Italy	0.63 (0.53–0.75)
Spain	0.62 (0.51–0.74)
France	0.59 (0.50–0.70)
Romania	0.55 (0.46–0.67)
Malta	0.46 (0.35–0.61)
Portugal	0.41 (0.33–0.51)

^a^adjusted for age, gender, education, physical activity level, general state of health, usual activity and country.

Table 3 shows the distributions of high-sit/low-active and low-sit/high-active groups across socio-demographic groups and countries. A larger proportion of the sample was classified as low-sit/high-active compared with high-sit/low-active (10.1% vs. 11.3%). Significant differences were observed within each sub-group across all sociodemographic factors and by country. There greater proportions of males than females in both the high-sit/low-active group and low-sit/high-active sub-group. Adults aged 65 years and over comprised the largest proportion of the high-sit/low-active sub-group. Adults who reported their general health as bad/very bad had the highest proportions in the high-sit/low-active group, and the lowest proportion in the low-sit/high-active group. A higher proportion of adults with ≥19 years of education were in the high-sit/low-active group, whereas a higher proportion of those with ≤18 years of education were found to be classified as low-sit/high-active. Across countries, although in some cases the sample sizes were small, the countries with the largest proportion of their sample within the high-sit/low-active group were Cyprus (Republic), Cyprus (TCC) and Croatia. Romania, Germany East and Bulgaria had the largest proportions within the low-sit/high-active group (Table 3).

**Table 3:** Percentage of sample classified as high sit/low-active^a^ or low-sit/high-active^b^ by sociodemographic factors and by country

	**High-sit/low-active**	**Low-sit/high-active**
	**% (n)**^**c**^	**% (n)**^**c**^
**All (% of total sample)**	10.1 (2 779)	11.3 (3 134)
**Gender**		
Male	9.9 (1 209)	14.7 (1 799)
Female	10.2 (1 570)	8.7 (1 335)
**Age**		
15 – 24 years	10.0 (376)	8.1 (304)
25 – 34 years	9.6 (430)	13.4 (602)
35 – 44 years	8.5 (418)	15.1 (746)
45 – 54 years	9.3 (421)	13.5 (607)
55 – 64 years	9.0 (397)	11.4 (502)
65 years and older	13.3 (737)	6.7 (373)
**Education**		
18 yrs and less of education	8.9 (1 342)	13.5 (2 046)
19 years and over of education	11.5 (1 069)	9.9 (923)
**General state of health**		
Very good	8.1 (465)	12.7 (731)
Good	9.0 (1 246)	11.5 (1 539)
Neither good nor bad	10.8 (635)	11.2 (660)
Bad/very bad	20.1 (423)	6.7 (142)
**Usual activity**		
Low sitting	5.2 (424)	18.3 (1 485)
Mixed sitting	11.7 (1 179)	7.1 (720)
High sitting	12.5 (1 176)	9.9 (929)
**Country**		
Cyprus (Republic)	21.2 (99)	9.4 (44)
Cyprus (TCC)	17.8 (74)	5.3 (22)
Croatia	14.2 (139)	15.4 (151)
Denmark	14.0 (137)	7.9 (77)
Belgium	14.0 (139)	9.3 (92)
Czech Republic	13.9 (131)	9.1 (86)
Great Britain	13.2 (119)	7.7 (69)
Northern Ireland	12.9 (37)	7.3 (21)
Luxembourg	12.4 (61)	9.6 (47)
Greece	12.3 (123)	9.3 (93)
Sweden	12.0 (124)	7.7 (79)
France	11.8 (115)	11.4 (111)
Ireland	11.2 (11.2)	8.4 (75)
Finland	11.0 (108)	9.6 (94)
Poland	10.2 (98)	12.1 (117)
Italy	10.1 (97)	10.7 (103)
Malta	9.9 (44)	3.8 (17)
Slovakia	9.1 (89)	15.0 (147)
Spain	8.9 (84)	7.1 (67)
Estonia	8.7 (83)	13.4 (127)
Germany West	8.6 (77)	11.3 (101)
Latvia	8.5 (85)	15.3 (153)
Austria	8.4 (84)	10.1 (101)
Turkey	7.7 (67)	5.7 (50)
Hungary	7.5 (73)	18.8 (182)
Slovenia	7.3 (73)	12.9 (130)
The Netherlands	6.9 (70)	7.6 (77)
Lithuania	6.2 (54)	16.2 (141)
Bulgaria	6.2 (57)	17.6 (163)
Romania	5.9 (58)	18.8 (184)
Germany East	5.7 (30)	19.4 (103)

^a^High-sit/low-active group calculated by being in the low active group (assessed by IPAQ) and the highest quartile of sitting group (420–960 mins/day).

^b^Low-sit/high-active calculated by being in the high active group (assessed by IPAQ) and the lowest tertile of sitting group (0–211 mins/day).

^c^ Percentages are given relative to the proportion of total number within each sociodemographic factor and country.

Multivariable-adjusted analyses for the odds of being in the high-sit/low-active or low-sit/high-active sub-groups are shown in Table 4. Adults who reported their general health as bad/very bad had the highest odds of being in the high-sit/low-active (OR = 4.74, CI_95_ = 3.97-5.65), while these adults were less likely to be classified as low-sit/high active (OR = 0.43, CI_95_ = 0.35-0.52). Compared to adults with 18 years or less of education, those with 19 years or more education (OR = 1.27, CI_95_ = 1.15-1.41) were more likely to be in the high-sit/low-active. Higher educated adults were less likely to be in the low-sit/high-active group (OR = 0.68, CI_95_ = 0.61-0.74). Across countries, compared with the average odds for the countries analysed, adults from Cyprus (Republic) (OR = 2.68, CI_95_ = 2.09-3.44) and Cyprus (TCC) (OR = 2.15, CI_95_ = 1.63-2.83) were more likely to be in the high-sit/low-active group, while those from East Germany (OR = 2.21, CI_95_ = 1.77-2.77) and Hungary (OR = 2.19, CI_95_ = 1.84-2.60) had the highest odds of being classified as low-sit/high-active.

**Table 4:** Adjusted odds ratios^a^ (OR) and 95% confidence intervals (95% CI) of belonging to high sit/low-active^b^ or low-sit/high-active^c^ by sociodemographic factors and by country

**Independent variables**	**High-sit/low-active**	**Low-sit/high active**
	**OR (95% CI)**	**OR (95% CI)**
**Gender (ref: male)**		
Female	0.99 (0.91–1.08)	0.50 (0.46–0.55)
**Age (ref: 15–24 years)**		
25 – 34 years	1.02 (0.81–1.27)	1.16 (0.97–1.39)
35 – 44 years	0.86 (0.69–1.08)	1.35 (1.13–1.61)
45 – 54 years	0.92 (0.73–1.15)	1.15 (0.96–1.38)
55 – 64 years	0.93 (0.74–1.18)	1.04 (0.86–1.26)
65 years and older	1.53 (1.19–1.96)	0.65 (0.52–0.81)
**Education (ref: 18 yrs and less of education)**		
19 years and over of education	1.27 (1.15–1.41)	0.68 (0.61–0.74)
**General state of health (ref: very good)**		
Good	1.44 (1.26–1.64)	0.78 (0.70–0.86)
Neither good nor bad	2.13 (1.83–2.48)	0.68 (0.60–0.78)
Bad/very bad	4.74 (3.97–5.65)	0.43 (0.35–0.52)
**Usual activity (ref: low sitting)**		
Mixed sitting	1.59 (1.35–1.86)	0.52 (0.45–0.60)
High sitting	2.87 (2.52–3.26)	0.48 (0.44–0.53)
**Country (ref: mean odds of all countries)**		
Cyprus (Republic)	2.68 (2.09–3.44)	0.85 (0.62–1.16)
Cyprus (TCC)	2.15 (1.63–2.83)	0.42 (0.27–0.65)
Belgium	1.65 (1.36–2.00)	0.89 (0.71–1.11)
Greece	1.60 (1.30–1.96)	0.78 (0.62–0.97)
Northern Ireland	1.57 (1.11–2.22)	0.59 (0.38–0.92)
Great Britain	1.56 (1.27–1.90)	0.66 (0.51–0.85)
Ireland	1.50 (1.19–1.89)	0.57 (0.45–0.74)
Denmark	1.49 (1.21–1.85)	0.80 (0.62–1.04)
Luxembourg	1.48 (1.11–1.98)	0.90 (0.66–1.23)
Croatia	1.42 (1.16–1.75)	1.65 (1.37–1.99)
Czech Republic	1.40 (1.15–1.70)	0.88 (0.70–1.11)
France	1.19 (0.97–1.47)	1.13 (0.92–1.39)
Sweden	1.19 (0.97–1.45)	0.79 (0.62–1.00)
Malta	1.17 (0.83–1.63)	0.28 (0.17–0.46)
Turkey	1.09 (0.81–1.45)	0.36 (0.27–0.49)
Italy	1.05 (0.83–1.32)	0.98 (0.79–1.22)
Finland	1.03 (0.83–1.28)	1.18 (0.94–1.48)
Poland	1.00 (0.80–1.25)	1.28 (1.04–1.58)
Spain	0.95 (0.74–1.23)	0.62 (0.48–0.80)
Austria	0.86 (0.67–1.09)	0.85 (0.68–1.05)
Germany West	0.82 (0.64–1.05)	1.09 (0.88–1.35)
Slovakia	0.77 (0.61–0.98)	1.66 (1.38–1.99)
Hungary	0.68 (0.53–0.87)	2.19 (1.84–2.60)
The Netherlands	0.65 (0.50–0.84)	0.74 (0.59–0.95)
Latvia	0.64 (0.50–0.82)	2.01 (1.67–2.42)
Estonia	0.63 (0.49–0.80)	1.85 (1.51–2.25)
Romania	0.60 (0.46–0.79)	1.91 (1.60–2.28)
Slovenia	0.59 (0.45–0.78)	1.60 (1.32–1.95)
Germany East	0.56 (0.38–0.82)	2.21 (1.77–2.77)
Bulgaria	0.54 (0.41–0.72)	1.85 (1.54–2.22)
Lithuania	0.47 (0.35–0.62)	2.06 (1.69–2.50)
Portugal	0.46 (0.33–0.64)	0.87 (0.70–1.09)
Cyprus (Republic)	2.68 (2.09–3.44)	0.85 (0.62–1.16)

^a^adjusted for age, gender, education, usual activity, general state of health and country.

^b^high-sit/low-active group calculated by being in the low-active group (assessed by IPAQ) and the highest quartile of sitting group (420–960 mins/day).

^c^low-sit/high-active calculated by being in the high-active group (assessed by IPAQ) and the lowest tertile of sitting group (0–211 mins/day).

## Discussion

This paper examined the prevalence and correlates of weekday sitting time among a large sample of European adults of the 32 Eurobarometer-participating countries. Population-based multi-country studies of sitting prevalence that use standardised instruments are scarce. Our study is the first, to our knowledge, to provide a comprehensive comparison of sitting prevalence across a large cross-section of European countries.

The key finding of our analysis of the Eurobarometer 64.3 was that IPAQ assessed usual weekday sitting time was geographically distributed, with countries within north-western Europe generally reporting higher sitting times than countries situated within south-eastern Europe (Figure 1). Within the scope of the present study it is possible only to speculate on the causes of this geographical pattern. However, an explanatory factor may be the unequal distribution of wealth between the more affluent north-western countries, when compared to less wealthy south-eastern Europe countries. Wealth inequalities could theoretically influence sitting time across all sitting domains (e.g. occupation, transport, leisure-time and household). Within the occupational setting, it is likely north-western countries have greater proportions of adults in white collar, office-based occupations (which require greater volumes of sitting), and greater exposure to technology at work (e.g. computers and other labour saving devices). Moreover, unequal wealth disruptions across Europe may result in greater proportions of passive transport (car use). For example, when compared to south-eastern Europe countries, north-western countries may be more likely to have building and transportation practices that require car use for most trips, [36]. Within the household domain, it is also possible that those within north-western countries have greater access to labour-saving devices and technology (e.g. internet, electronic entrainment) [36]. Although these data were collected in a similar time-frame (November-December 2005), it is possible that climatic factors may partially explain differences in sitting times across countries. For example, northern European countries tend to be in the highest quartile of sitting time, where the climate is colder than in southern European countries where fewer people belonged to the highest quartile. Furthermore, cultural differences in interpreting sitting questions and reporting biases may have contributed to some of the differences in sitting time between countries [37].

The present study showed that usual weekday sitting time among 32 European countries was 309 minutes/day. This daily sitting time was slightly lower than what was observed by the IPS which reported a mean sitting time of 346 minutes/day [28]. When compared to population studies using objective assessment instruments, sitting time may be underestimated by self-report measurements [38,39]. For example, sitting data from the IPS and Eurobarometer 64.3 indicated that adults self-reported 5–6 hrs of sitting per day. However, accelerometer data from several national population representative samples has shown daily sedentary time to be 8.8-11.2 h/day [13,40-42]. Despite the disparity between objective and subjective tools, it is not clear whether the prevalence of sitting observed in this study are harmful to health because at present there is no consensus among researchers surrounding the dose–response relationship between sitting and detrimental health outcomes [43,44]. In parallel with the wide variations in physiological adaptations to exercise training [45], it is likely that the dose and volume of sitting and associated health consequences differs from individual to individual [45,46]. Moreover, the way sitting time is accrued within the context of a whole day may be important [47]. Recently, controlled laboratory studies further examined the acute cardiometabolic effects of breaking up prolonged sedentary time [47,48]. In these experiments, when prolonged sitting was displaced with light- or moderate-intensity walking, there was a significant lowering of postprandial glucose and insulin levels [47], and improved whole-body insulin sensitivity [48].

The adjusted analyses identified that being low-active, being in poor health and having high sitting usual activities were among the strongest correlates of reporting high sitting time. These findings are somewhat similar to other studies [28,33,34]. For education, our results and those from the IPS [28], suggest that high levels of education are associated with higher levels of sitting. This result may be explained by the fact that those with higher education levels may have occupations that require higher volumes of sitting. Although we assessed sitting time in a somewhat younger population than the IPS study (EB 64.3: ≥15 years vs. IPS: ≥18 years), both studies show a trend for younger people to report higher sitting times. Potential explanations may be that in contrast to older adults, a greater proportion of younger adults may be students, which may require high sitting volumes [28,49,50]. Moreover, despite younger adults having a greater amount of leisure time, time use studies have shown that 85-90% of their leisure time is spent sedentary [49,50]. Last, younger adults may be less likely to household activities (which are often not sedentary) [50]. These self-report data on sitting time and its relationship to age are to some extent inconsistent with studies using objective physical activity assessment tools. For example, in the U.S. population NHANES 2003–2004 study, accelerometer-defined sedentary time showed a linear trend, with sitting times increasing with increasing age [13]. However, a potential explanation of these inconsistent findings may be the age differences of the participants assessed in the EB 64.3 (15-18 years) and NHANES 2003–2004 participants (>20 years). A recent review of population studies assessing accelerometer-defined sedentary time of Belgian adults and children (n = 2,083) showed that those aged between15-18 years had the highest levels of sedentary time when compared to other age groups [51]. These conflicting findings highlight the need for further research that objectively assesses cross-country sedentary time within population-representative samples and among a wide variety of age groups.

The findings around sitting time and physical activity levels are consistent with previous research, with the IPS study showing a similar inverse relationship between sitting time and levels of physical activity [28]. This finding differs from some small-scale research using objective assessments of physical activity and sedentary behaviour patterns. For example, when accelerometer-assessed physical activity and sedentary time were examined among participants in short-term exercise studies, those engaging in high levels of moderate-to-vigorous exercise may compensate for high activity levels by being more sedentary during non-exercise periods [52,53], resulting in no net gain in energy expenditure [54]. While the present study does not support this hypothesis, it may be possible that adults reporting high physical activity levels may under report their sitting time. The possible compensation for increased sitting time among adults with high levels of physical activity warrants further investigation in population-representative samples. Furthermore, objective assessments tools, such as accelerometers and inclinometers, should be used to examine relationships between sitting and physical activity behaviour patterns. However, population studies that implement objective physical activity assessment tools have significant cost and logistical issues [13].

A novel aspect of this study was the description of the high-sit/low-active and low-sit/high-active specific sub-groups within the Eurobarometer 64.3 sample, which reflects the combined risks of sitting and levels of physical activity. The cross-country distributions of these sub-groups suggested another potential geographical pattern. There was a greater tendency for adults from north-western European countries to be classified as high-sit/low-active. In contrast, those within south-eastern countries were more likely to be classified as low-sit/high-active. While this trend requires replication, this geographical pattern suggests that adults within north-western European countries may be at risk of health consequences associated with a combination of high volumes of sitting and low levels of physical activity. Another observation was that some countries that scored high on sitting prevalence (Netherlands, Denmark, West-Germany), scored relatively low in the high-sit/low-active category. This seems to indicate higher levels of physical activity in these countries, which might be partly due to the good active transportation infrastructure in these countries. In contrast, Great Britain which has poorer active transportation infrastructure scored much higher on the high-sit/low-active category. More educated adults were classified more frequently as high-sit/low-active, and less frequently as low-sit/high-active. These findings differ from previous research examining physical activity levels without relation to sitting time. Research has consistently shown a positive association between increased education levels and high physical activity levels [23,55]. Although the analyses adjusted for usual activities, this finding may be explained by higher educated adults employed in occupations that require higher volumes of sitting. Future studies should continue to assess sitting and physical activity patterns concurrently. Research examining the prevalence and correlates of high-sit/low-active individuals may be used to target at risk populations in intervention studies.

Strengths of this study include the recruitment of a large sample of adults across a large number of European countries. This resulted in a reasonably heterogonous sample, making it possible to compare sitting data across various sociodemographic factors. A further strength was the use of a standardised sitting time assessment instrument, which makes it possible to compare the findings of the present study to future research. Limitations include the cross-sectional design, which makes it difficult to infer causality from the study findings. Moreover, given that the modest response rate of 54.6%, there may be limitations around the generalisability of these results. Further limitations included the use of self-report measures of sitting and physical activity, which may result in social desirability and recall biases [56]. Furthermore, it is also possible that those who participated in the study may have different sitting patterns than non-participants. Therefore, these factors might have resulted in the under-reporting of sitting time, which may suggest our estimates of sitting time in Europe are on the conservative side. However, given the large sample size across a wide-variety of countries, objective sitting and physical activity assessments (e.g. accelerometers and inclinometers) were too difficult to implement due to high cost, logistical issues and participant burden. Furthermore, despite limitations around the validity of self-reported methods, among large samples, standardised self-report tools have a use for ranking individuals sitting time and physical activity levels [57].

## Conclusion

With emerging evidence suggesting that high volumes of sitting may be an independent health risk factor, there is a need to describe the prevalence of population levels of sitting. However, large-scale multi-country sitting surveillance studies are scarce. The Eurobarometer 64.3 survey provided a unique opportunity to examine the prevalence of sitting time among a population sample of adults from 32 European countries. This paper identified a large variation in sitting time across European countries, with indications that populations in north-western Europe sit the most. Moreover, high sitting volumes were more prevalent among males, younger age groups, low-active adults, and those with higher education levels. Given the recent trend for technological and societal advancements across developed and developing countries, sitting is, and will become increasingly entrenched in many modern lifestyles. Regular public health surveillance studies using consistent methods and survey instruments on population sitting levels should be a priority for national and international health departments.

## Consent

The European Commission approved study protocols and written informed consent was obtained from the participants for the publication of this report [35].

## Competing interests

The authors declare that they have no competing interests.

## Authors’ contributions

JB designed the study, developed the research plan and conducted the preliminary data reduction/analysis and wrote the manuscript. AB and JC provided guidance on the analysis and interpretation of data. AD and JB conducted the logistic regression analysis. MS and HvdP critically reviewed the manuscript and interpretation of the results. All authors read and approved the final version of the manuscript for publication.
